# Multiple contiguous cervicothoracic Clay-shoveler's fractures (From C6 to T9 spinal vertebrae)

**DOI:** 10.11604/pamj.2013.16.128.3531

**Published:** 2013-12-02

**Authors:** Ali Akhaddar, Cherkaoui Mandour

**Affiliations:** 1 Department of Neurosurgery, Avicenne Military Hospital, Marrakech, Morocco; 2 Department of Neurosurgery, Mohammed V Military Teaching Hospital, University of Mohammed V Souissi, Rabat, Morocco

**Keywords:** Clay-shoveler fractures, vertebra, spine

## Image in medicine

Clay-shoveler's fracture is an avulsion fracture of one or more spinous processes of the lower cervical and/or upper thoracic vertebra. The site of fracture is most commonly C6, C7 or T1 but it occurs rarely at multiple levels. More often the mechanism is a combination of direct impact and hyperflexion of the neck. It concerns healthy individuals with no history of prior disease. These injuries are known to be stable but painful at the cervicothoracic site without neurological symptoms. In most patients, immobilization of the neck with a cervical collar and restriction of physical activity for 1 to 2 months frequently result in pain relief. This 29-year-old woman was admitted following a road traffic accident. She had severe neck and posterior thoracic pain with radiation to the bilateral shoulder regions. Physical examination revealed tenderness over the posterior cervicothoracic spine without neurological deficit. Computed Tomography (CT) scan of the spine revealed isolated fractures of the spinous processes interesting eleven adjacent levels from C6 to T9 spinal vertebrae without any other traumatic bony lesions. Conservative treatment was administered (analgesic therapy and muscle relaxant) and immobilization was maintained for 6 weeks followed by a good outcome.

**Figure 1:**
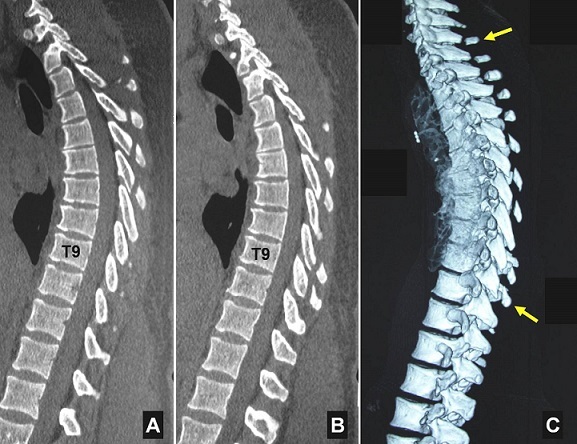
A, B) spinal sagittal cervicothoracic ct-scan with 3d bony reconstruction; C)contiguous isolated fractures of the spinous processes from C6 to T9 spinal vertebrae

